# Slowly progressive interstitial lung disease preceding typical dermatomyositis symptoms in anti-melanoma differentiation-associated gene 5 antibody-positive clinically amyopathic dermatomyositis

**DOI:** 10.1016/j.rmcr.2021.101491

**Published:** 2021-07-27

**Authors:** Taisuke Isono, Hiromi Nakajima, Kenji Takano, Yoichi Kobayashi, Yoshinori Kawabata, Yoshihiko Shimizu, Noboru Takayanagi

**Affiliations:** a Department of Respiratory Medicine, Saitama Cardiovascular and Respiratory Center, Saitama, Japan; b Departments of Pathology, Saitama Cardiovascular and Respiratory Center, Saitama, Japan

**Keywords:** Anti-melanoma differentiation-associated gene 5, Clinically amyopathic dermatomyositis, Interstitial lung disease, Slowly progressive, Surgical lung biopsy, ANCA, cytoplasmic autoantibody, BAL, bronchoalveolar lavage, CADM, clinically amyopathic dermatomyositis, CMV, cytomegalovirus, CT, computed tomography, DM, dermatomyositis, HRCT, high-resolution computed tomography, ILD, interstitial lung disease, IVCY, intravenous cyclophosphamide, KL-6, Krebs von den Lungen-6, MDA5, anti-melanoma differentiation-associated gene 5, PaO_2_, partial pressure of oxygen, PSL, prednisolone, RP-ILD, rapidly progressive interstitial lung disease, SLB, surgical lung biopsy, TBLB, transbronchial lung biopsy

## Abstract

A 73-year-old woman who visited our hospital complaining of dry cough for three months was refractory to antimicrobial therapy. Chest computed tomography revealed subpleural consolidation. Specimens obtained from surgical lung biopsy revealed subpleural perilobular airspace organization and fibrosis. After the biopsy, mechanic's hand and Gottron's papules appeared, and anti-melanoma differentiation-associated gene 5 (MDA5) antibody was found to be positive. Subsequently, anti-MDA5 antibody measured in cryopreserved serum from her first admission proved to be positive. It is difficult to suspect the presence of anti-MDA-5 antibody in patients with interstitial lung disease without typical dermatomyositis symptoms or slow disease progression.

## 1 Introduction

Dermatomyositis (DM) is an inflammatory muscle disease accompanied by skin lesions and, frequently, interstitial lung disease (ILD). Clinically amyopathic DM (CADM) is a subset of DM characterized by typical skin lesions but no clinical muscle weakness or elevation of myogenic enzymes [1]. Several studies showed that anti-melanoma differentiation-associated gene 5 (MDA5) antibody was detected in 12.0%–32.0% of DM patients, and it was associated with CADM and rapidly progressive ILD (RP-ILD) [[2], [3], [4], [5]]. The mortality rate of ILD complicated by anti-MDA5-positive DM was reported to be 30.3%–54.5% [[6], [7], [8], [9]].

Some patients with anti-MDA5 antibody develop ILD preceding typical DM symptoms. Moreover, patients with anti-MDA5 antibody who develop slowly progressive ILD were also reported, but in such patients, it is difficult to suspect the presence of anti-MDA5 antibody. Although, pathological findings of anti-MDA5 antibody-positive ILD have been reported, most of the patients were evaluated by autopsy. We report a woman with anti-MDA5 antibody-positive CADM who developed slowly progressive ILD preceding typical DM symptoms and underwent surgical lung biopsy (SLB) to evaluate the ILD pathologically.

## 2 Case presentation

A 73-year-old woman visited a local doctor complaining of persistent dry cough for three months. Chest computed tomography (CT) showed consolidation in the right lower lobe. She was considered to have bacterial pneumonia and received treatment with garenoxacin for one week, but her symptoms did not improve. Then, she was referred to our institution where she was diagnosed as having diabetes mellitus at 73 years old and received treatment with sitagliptin 50 mg/day. She had no history of smoking or dust exposure.

Her vital signs on the first visit included a heart rate of 73 beats/min, blood pressure of 110/63 mmHg, and body temperature of 35.9 °C. Chest auscultation revealed fine crackles. She had no signs or symptoms of edema, arthritis, skin lesions characteristic of DM, myalgia, muscle tenderness, or muscle weakness.

**Fig. 1:**
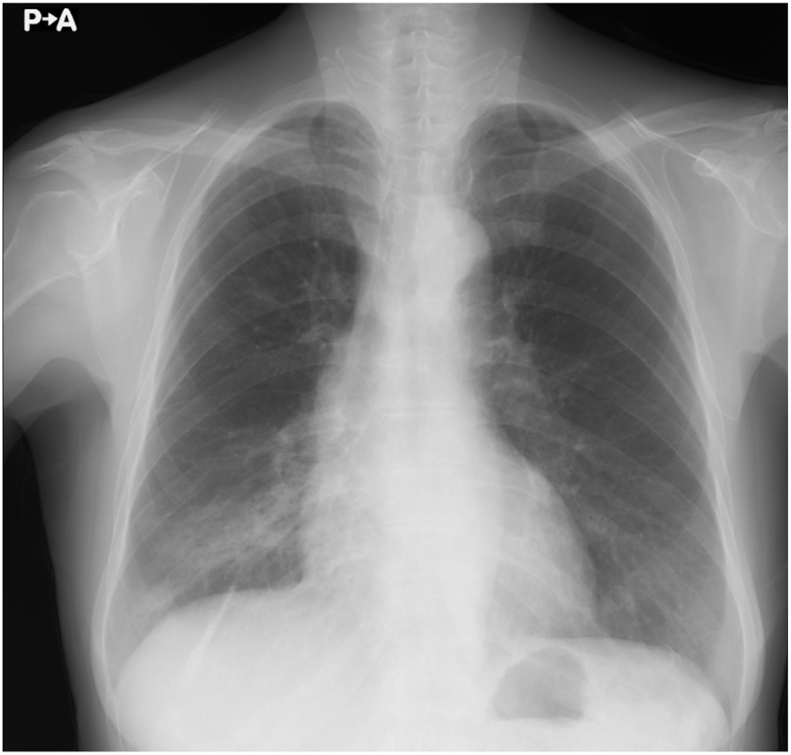
Chest radiography revealed consolidation in the right lower lung field at the initial visit.

**Fig. 2:**
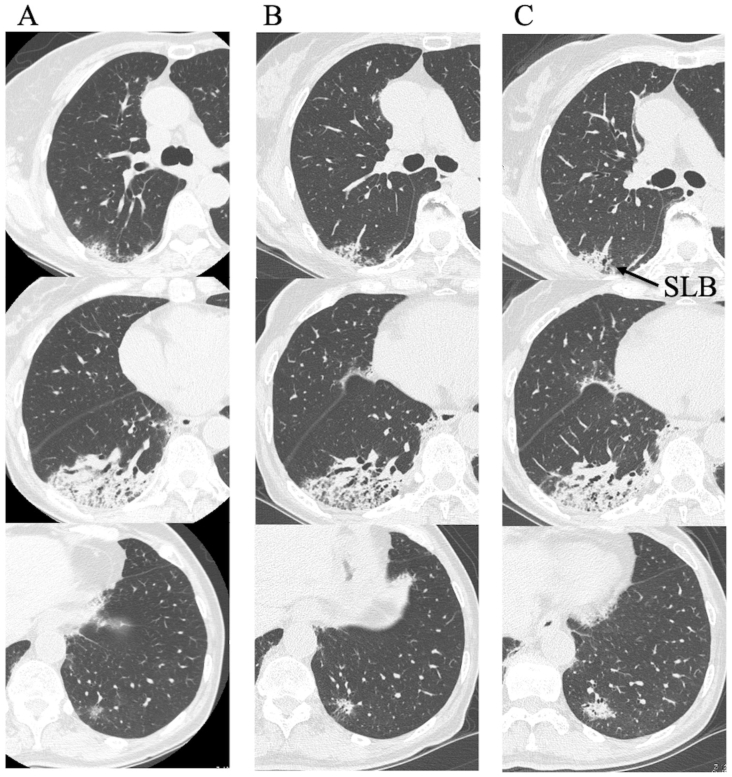
Pulmonary findings of chest high-resolution computed tomography. (A) At the initial visit, subpleural consolidation with volume reduction in the right lower lobe and localized ground-glass opacity in the left lower lobe were present. (B) Two weeks after she began receiving prednisolone (PSL), consolidation had not improved. (C) Two months after discontinuation of PSL, bilateral consolidation developed. Then, a surgical lung biopsy (SLB) of the right upper lobe was performed (arrow).

Arterial blood gas analysis under ambient air showed a pH of 7.423, partial pressure of carbon dioxide of 39.7 mmHg, and partial pressure of oxygen (PaO_2_) of 74.3 mmHg. Laboratory findings were as follows: white blood cell count, 6300/mm^3^; neutrophils, 4300/mm^3^; eosinophils, 200/mm^3^; basophils, 0/mm^3^; monocytes, 400/mm^3^; lymphocytes, 1300/mm^3^; hemoglobin, 11.9 g/dL; platelet count, 26.3 × 10^4^/mm^3^; serum total protein, 7.3 g/dL; albumin, 4.0 g/dL; normal liver transaminase; lactate dehydrogenase, 207 IU/L; creatine kinase, 72 U/L; creatinine, 0.68 mg/dL; C-reactive protein, 0.26 mg/dL; ferritin, 471 ng/mL; Krebs von den Lungen-6 (KL-6), 609 U/mL; surfactant protein-D, 42.0 ng/mL; and brain natriuretic peptide, 7.9 pg/mL. Her anti-nuclear antibody titer was × 40 with a homogeneous pattern. Rheumatoid factor, anti-cyclic citrullinated peptide antibody, anti-aminoacyl tRNA synthetase antibody, anti-Sjögren's-syndrome-related antigen A antibody, proteinase 3-anti-neutrophil cytoplasmic autoantibody (ANCA), and myeloperoxidase ANCA were all negative, as were beta-D glucan, *Cryptococcus* antigen, and interferon gamma release assay (QuantiFERON®). Pulmonary function testing showed obstructive respiratory dysfunction (Table 1). Chest radiography showed consolidation in the right lower lung field (Fig. 1). Chest high-resolution CT (HRCT) showed subpleural consolidation with volume reduction in the right lower lobe and localized ground-glass opacity in the left lower lobe (Fig. 2A). Bronchoalveolar lavage (BAL) obtained from the right B^8b^ had a total cell count of 1.7 × 10^5^ cells/mL (macrophages, 45.0%; lymphocytes, 48.1%; neutrophils, 2.3%; and eosinophils, 4.6%). The CD4/CD8 lymphocyte ratio was 2.2. No significant pathogens were cultured from the BAL. Transbronchial lung biopsy (TBLB) of the right B^8a^ was performed, but no alveoli were present in the specimens.

**Table 1:** Pulmonary function test results.

Parameter	Result	
VC	3.16	L
VC, %predicted	113.3	%
FVC	3.04	L
FVC, %predicted	109.0	%
FEV1	2.1	L
FEV1, %predicted	131.3	%
FEV1/FVC	69.1	%
TLC	4.9	L
RV	1.73	L
RV, %predicted	137.3	%
RV/TLC	35.3	%
DLCO	15.11	mL/min/mmHg
DLCO, %predicted	98.6	%
DLCO/VA	3.08	mL/min/mmHg/L
DLCO/VA, %predicted	71.5	%

DLCO, diffusing capacity of the lungs for carbon monoxide; FEV1, forced expiratory volume in 1 second; FVC, forced vital capacity; RV, residual; TLC, total lung capacity; VA, alveolar gas volume; VC, vital capacity.

**Fig. 3:**
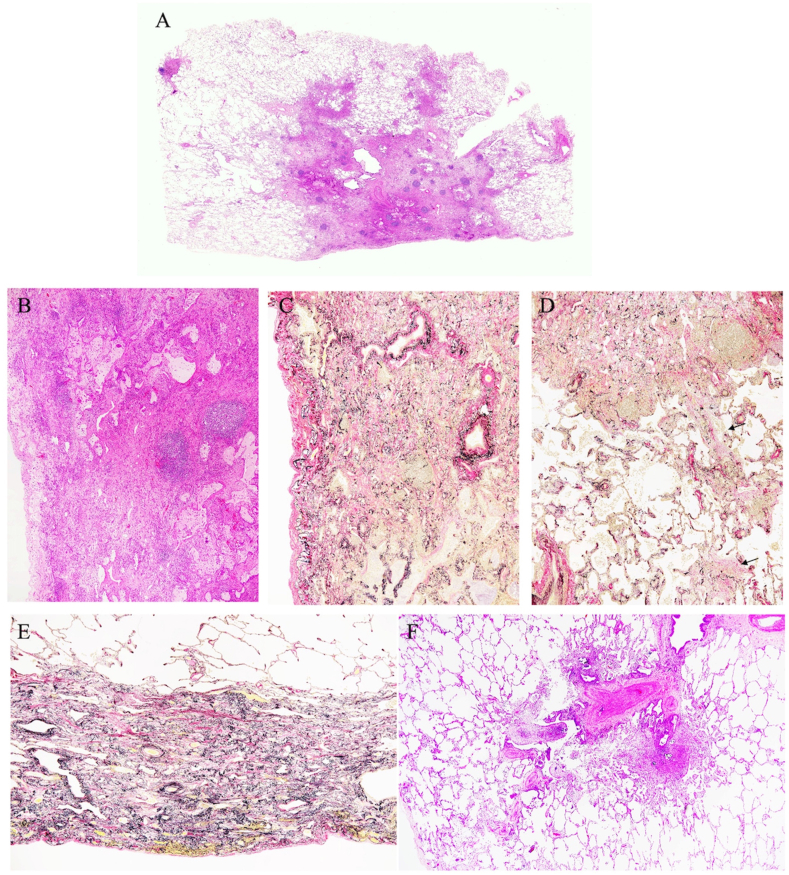
Pathological findings of the surgical lung biopsy. (A) Subpleural perilobular airspace organization and fibrosis (hematoxylin and eosin [HE] staining; panoramic view). (B) Many lymphocytic and plasmocytic infiltrates and lymphatic follicles were present in the lesion. (HE staining; magnification, × 400). (C, D) The alveolar structure was preserved and mural organization was present (Elastica van Gieson staining; magnification, × 400). (E) Subpleural fibroelastosis-like pleuroparenchymal fibroelastosis (HE staining; magnification, × 400). (F) Inflammation in the respiratory bronchioles (HE staining; magnification, × 400).

We suspected that she might have cryptogenic organizing pneumonia, invasive mucinous adenocarcinoma, or malignant lymphoma. We treated her with prednisolone (PSL) 40 mg per day and observed the response to the treatment. Although she received PSL for two weeks, her pulmonary lesions did not improve (Fig. 2B). We discontinued the PSL and performed TBLB of the right B^10^, but only a few atypical cells were present in the specimens. Two months after discontinuation of the PSL, chest HRCT showed slight worsening of the left lower lobe consolidation (Fig. 2C). A SLB of the right upper lobe was performed to obtain a definitive diagnosis. The specimen showed subpleural perilobular airspace organization and fibrosis, subpleural fibroelastosis, and inflammation in the respiratory bronchioles (Fig. 3). We considered that she had ILD, but we could not specifically diagnose ILD based on these pathological findings. Therefore, we determined that she had unclassifiable idiopathic interstitial pneumonia.

**Fig. 4:**
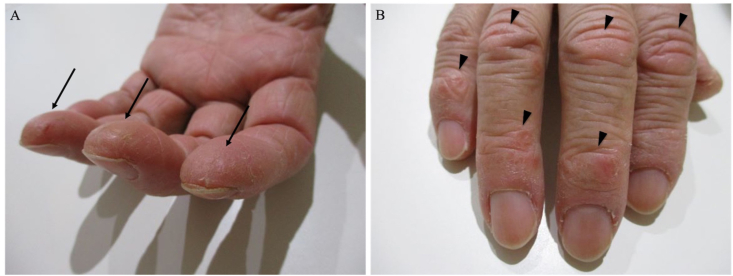
(A) Mechanic's hand (arrows). (B) Gottron's papules on the dorsum (arrowheads).

**Fig. 5:**
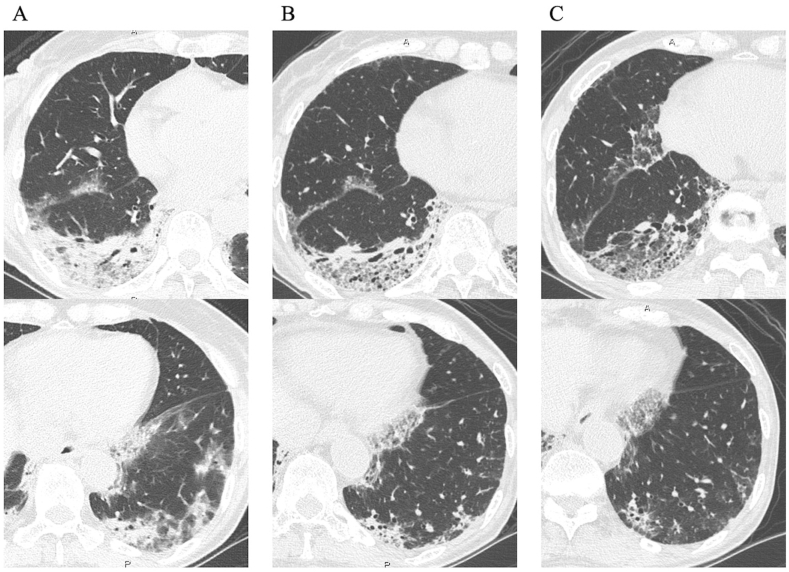
Pulmonary findings of chest high-resolution computed tomography. (A) One month after the surgical lung biopsy, bilateral subpleural consolidation and ground-glass opacity had worsened. (B) One month and (C) six months after starting the treatment, these findings had improved.

**Fig. 6:**
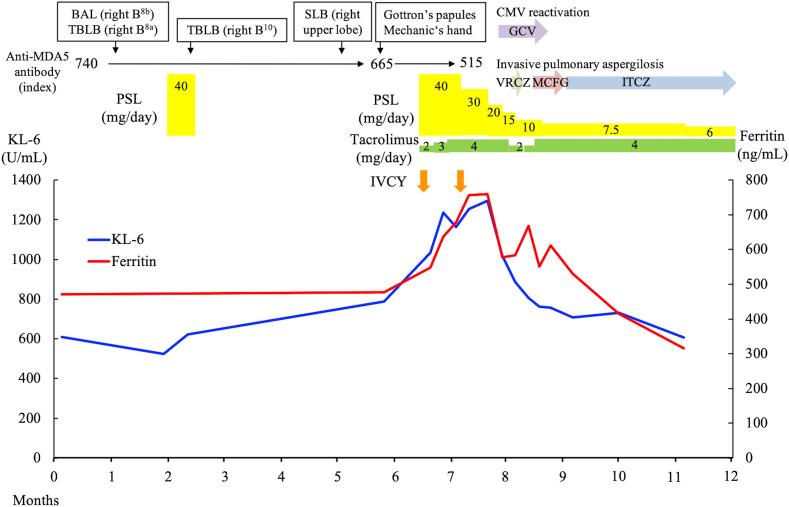
Clinical course of the patient. BAL: bronchoalveolar lavage, CMV: cytomegalovirus, GCV: ganciclovir, ITCZ: itraconazole, IVCY: intravenous cyclophosphamide, KL-6: Krebs von den Lungen-6, MCFG: micafungin, MDA-5: melanoma differentiation-associated gene 5, PSL: prednisolone, SLB: surgical lung biopsy, TBLB: transbronchial lung biopsy, VRCZ: voriconazole.

One month after SLB (6 months after the first visit), cracking and hyperkeratosis of the tips and margins of her fingers (mechanic's hand) and erythematous papules on the dorsum of the right proximal interphalangeal joints and distal interphalangeal joints (Gottron's papules) appeared (Fig. 4). Manual muscle testing revealed no weakness of the muscles, and electromyography showed no myogenic changes. Arterial blood gas analysis under ambient air showed a PaO_2_ of 78.1 mmHg. Her levels of lactate dehydrogenase of 270 U/L, KL-6 of 789 U/mL, surfactant protein-D of 130 ng/mL, ferritin of 477 ng/mL, and C-reactive protein of 1.62 mg/dL were all elevated. The anti-MDA5 antibody index was 665 (normal: <32). Her levels of creatine kinase of 51 U/L, myoglobin of <21 ng/dL, and aldolase of 5.2 IU/L were not elevated. Chest HRCT showed worsening of the bilateral subpleural consolidation and ground-glass opacity (Fig. 5A). On the basis of these findings, we held a multidisciplinary discussion and made a diagnosis of anti-MDA5 antibody-positive CADM associated with ILD. She was then treated with PSL, tacrolimus, and intravenous (IV) cyclophosphamide, and she was administered trimethoprim/sulfamethoxazole as prophylaxis for pneumocystis pneumonia. IV cyclophosphamide was discontinued after two cycles because cytomegalovirus (CMV) infection and invasive pulmonary aspergillosis developed. After the treatment was initiated, her clinical symptoms improved, and her KL-6 and ferritin levels decreased (Fig. 6). Six months after starting the treatment, chest HRCT showed improvement of the consolidation and ground-glass opacity (Fig. 5B and C). Subsequently, the anti-MDA5 antibody index was measured in cryopreserved serum obtained on her first admission, and it proved to be 740.

## 3 Discussion

We experienced a patient with anti-MDA5 antibody-positive CADM associated with slowly progressive ILD preceding DM symptoms. Initially, we suspected that this patient had cryptogenic organizing pneumonia, invasive mucinous adenocarcinoma, or malignant lymphoma, but her clinical course, pathological findings, and presence of anti-MDA5 antibody led to the diagnosis of ILD associated with CADM.

Patients with anti-MDA5 antibody-positive DM complicating ILD preceding DM symptoms have been reported. Tamai et al. reported a patient with anti-MDA5 antibody-positive CADM who developed skin lesions one month after the onset of RP-ILD [10]. Hamaguchi et al. analyzed 43 patients with anti-MDA5-positive DM and reported that two patients (4.7%) had ILD as the initial symptom [11]. Moreover, several patients with anti-MDA5 antibody-positive RP-ILD without DM symptoms were also reported [[12], [13], [14]]. All were started on immunosuppressive treatment at the diagnosis of RP-ILD, but most of them died within one to two months from the onset of the ILD. Therefore, they might not have had symptoms of DM other than ILD.

Similar to the present case, several patients with DM who developed slowly progressive ILD were reported. Huang et al. investigated 21 Canadian patients with anti-MDA5-positive DM and reported that they all had ILD. Of these patients, 6 (28.6%) had asymptomatic ILD, and 7 (33.3%) had symptomatic and chronic ILD [15]. Tsuji et al. studied 44 Japanese patients with anti-MDA5-positive DM who developed ILD and reported that 9 of them (20.5%) had chronic ILD [16].

Pathological findings of anti-MDA5 antibody-positive ILD were also reported. In most of the reported cases, autopsy was performed, and the pathological pattern of ILD was that of diffuse alveolar damage [[17], [18], [19]]. Chino et al. reported a patient with anti-MDA5 antibody-positive ILD who underwent SLB. The pathological findings of the patient indicated a diffuse alveolar damage pattern [20]. There were no reports of patients diagnosed as having unclassifiable idiopathic interstitial pneumonia pattern based on SLB or autopsy in anti-MDA5 antibody-positive ILD, as in the present case.

Although there is no international consensus on the treatment of anti-MDA5 antibody-positive ILD, the efficacy of treatment with corticosteroid and immunosuppressants has been reported. In the report by Tsuji et al., these authors compared combined immunosuppressive therapy comprising high-dose corticosteroid, tacrolimus, and IVCY with step-up treatment comprising corticosteroid and stepwise addition of immunosuppressants [16]. The combined immunosuppressive regimen group had both higher 6-month survival rates than the step-up treatment group (89% vs. 33%) but also more frequent CMV infection (85% vs. 33%). Besides, Sugiyama et al. investigated 116 patients with ILD complicated by DM or polymyositis and revealed that combination immunosuppressive treatment including corticosteroid, IVCY, and calcineurin inhibitor carried a high risk for serious infection [21]. The present patient also developed invasive pulmonary aspergillosis and CMV infection after starting a combined immunosuppressive regimen. Considering the efficacy of the combined immunosuppressive regimen, appropriate assessment and management of infection is necessary.

In conclusion, we encountered slowly progressive ILD in a patient with anti-MDA5 antibody-positive CADM. It is difficult to suspect the presence of anti-MDA-5 antibody in patients with preceding ILD that does not present an acute or subacute onset. The present patient also developed CMV infection and invasive pulmonary aspergillosis. Appropriate assessment and management of infection is necessary during administration of a combined immunosuppressive regimen.

## Funding

This research did not receive any specific grant from funding agencies in the public, commercial, or not-for-profit sectors.

## Declarations of competing interest

None.
